# Influence of Arctic sea-ice loss on the Greenland ice sheet climate

**DOI:** 10.1007/s00382-021-05897-4

**Published:** 2021-07-29

**Authors:** Raymond Sellevold, Jan T. M. Lenaerts, Miren Vizcaino

**Affiliations:** 1 Department of Geoscience and Remote Sensing, Delft University of Technology, Delft, The Netherlands; 2 Department of Atmospheric and Oceanic Sciences, University of Colorado Boulder, Boulder, CO USA

**Keywords:** Arctic, Sea-ice, GrIS Surface mass balance, Community Earth System Model 2.1

## Abstract

The Arctic is the region on Earth that is warming the fastest. At the same time, Arctic sea ice is reducing while the Greenland ice sheet (GrIS) is losing mass at an accelerated pace. Here, we study the seasonal impact of reduced Arctic sea ice on GrIS surface mass balance (SMB), using the Community Earth System Model version 2.1 (CESM2), which features an advanced, interactive calculation of SMB. Addressing the impact of sea-ice reductions on the GrIS SMB from observations is difficult due to the short observational records. Also, signals detected using transient climate simulations may be aliases of other forcings. Here, we analyze dedicated simulations from the Polar Amplification Model Intercomparison Project with reduced Arctic sea ice and compare them with preindustrial sea ice simulations while keeping all other forcings constant. In response to reduced sea ice, the GrIS SMB increases in winter due to increased precipitation, driven by the more humid atmosphere and increasing cyclones. In summer, surface melt increases due to a warmer, more humid atmosphere providing increased energy transfer to the surface through the sensible and latent heat fluxes, which triggers the melt-albedo feedback. Further, warming occurs throughout the entire troposphere over Baffin Bay. This deep warming results in regional enhancement of the 500 hPa geopotential heights over the Baffin Bay and Greenland, increasing blocking and heat advection over the GrIS’ surface. This anomalous circulation pattern has been linked to recent increases in the surface melt of the GrIS.

**Supplementary Information:**

The online version contains supplementary material available at 10.1007/s00382-021-05897-4.

## Introduction

Arctic amplification, the rapid warming of the Arctic relative to global warming, is a prominent sign of recent climate change that has emerged in the late 1990s (Serreze and Francis 2006; Serreze et al. 2009). A combination of many factors causes warming in the Arctic, e.g., atmospheric transport of heat from the midlatitudes (Screen et al. 2012), trapping of longwave radiation by CO$_{2}$ (Pithan and Mauritsen 2014), increased water vapor, and albedo-temperature-feedback due to thinning and retreat of sea ice (Screen et al. 2012; Pithan and Mauritsen 2014).

The GrIS is the largest freshwater body in the Arctic region and would raise the global mean sea level by 7.4 m if melted entirely (Bamber et al. 2018a). Since 2012, the GrIS has been losing mass at a rate of 247 Gt year$^{-1}$ (0.69 mm year$^{-1}$ of global sea-level rise; Bamber et al. 2018b), after the ice sheet being in an approximate mass balance before the 1990s (Hanna et al. 2008). An increase in the melt of the Greenland ice sheet (GrIS) follows the onset of Arctic warming (Trusel et al. 2018).

Surface mass balance (SMB) decline is the primary contributor ($\sim$60%) to the current GrIS mass loss, with recent increases in ice discharge as the second contributor ($\sim$40%) (van den Broeke et al. 2016). The GrIS surface gains mass through snowfall, rainfall that refreezes in the snow, and through deposition/riming (Ettema et al. 2010). On the other hand, the surface loses mass through melt that is not refrozen within the snow layers and sublimation. Melt occurs at low elevations when the ice sheet’s temperature reaches 0 $^{\circ }$C, and there is a surplus of energy (van den Broeke et al. 2008). As a result, the SMB of the GrIS has a strong seasonal cycle, with net mass gain in fall, winter, and spring, and net mass loss during the summer months (Vizcaino et al. 2013; Ran et al. 2018).

While sea ice loss and its impacts on high latitude climate have been extensively investigated (Francis and Vavrus 2012; Screen et al. 2013b; Barnes and Screen 2015a; Barnes and Polvani 2015b), little attention has been paid to the potential influence on the GrIS SMB. Observational studies suggest sea ice loss has a small impact on summer melt at the GrIS surface, restricted to western low-elevation areas (Rennermalm et al. 2009; Liu et al. 2016; Stroeve et al. 2017). When sea ice loss occurs close to the GrIS, the atmosphere becomes warmer and moister due to increased contact with the open ocean, leading to increased incoming longwave radiation at the surface of the ice sheet. More incoming longwave warms the surface and leads to increased melt. The melt attributable to increased turbulent heat fluxes over the GrIS surface is small, as katabatic winds block the onshore flow over the GrIS. However, the non-zero contribution might suggest a barrier wind mechanism (van den Broeke and Gallée 1996) mixing the onshore winds with the offshore katabatic winds (Stroeve et al. 2017). In years with an extensive melt of both Arctic sea ice and the GrIS, anomalous atmospheric ridging occurs over the GrIS (Liu et al. 2016). While this circulation pattern is likely not caused by sea ice loss, the sea ice loss may reinforce this circulation pattern (Rennermalm et al. 2009). Modeling studies (Day et al. 2013; Noël et al. 2014; Liu et al. 2016) corroborate the observational evidence of an impact of sea ice loss on the GrIS. Additionally, Liu et al. (2016) find that the anomalous ridging induced by sea ice loss can lead to an increase in summer atmospheric blocking events. Blocking events are caused by quasi-stationary synoptic high-pressure systems that block the westerly flow. Further, Noël et al. (2014) find that annual precipitation in the southeast of the GrIS increases in response to reduced sea ice.

The short observational record makes it challenging to detect robust mechanisms linking sea ice loss with increased GrIS surface melt due to the large interannual variability and their co-relationship with global warming. Also, available modeling studies are short (5 years), lack a physical calculation of GrIS SMB, and/or cannot capture potential large-scale atmospheric circulation changes. Motivated by these gaps, we use the Community Earth System Model version 2.1 (CESM2) to simulate the climate response to Arctic sea ice and sea surface temperature (SST) perturbations to determine the response of the GrIS SMB to ongoing and future sea ice loss, and understand the underlying processes. CESM2 features a physically based calculation of SMB, making it a state-of-the-art framework to assess the potential impacts of ongoing/future reductions in Arctic sea ice on GrIS SMB. To increase the robustness of our results, we analyze large ensembles (100 members per experiment).

## Methods

### Model

We use the Community Earth System Model 2.1 (CESM2; Danabasoglu et al. 2020). This model is a participant in the climate model intercomparison project (CMIP) phase 6 (Eyring et al. 2016). The model was run with active atmosphere, sea ice, land, ice sheet components, and prescribed ocean sea surface temperatures (SSTs). The atmospheric model is the Community Atmosphere Model version 6 (Gettelman et al. 2019), run at a horizontal resolution of 1.25$^{\circ }$ (longitude) $\times$ 0.9$^{\circ }$ (latitude) and employing 32 vertical levels. The sea ice model is the Los Alamos Sea Ice Model version 5 (CICE5; Hunke et al. 2017), run at a nominal 1$^{\circ }$ resolution with prescribed sea ice concentrations (SICs). The land model is the Community Land Model version 5 (CLM5; Lawrence et al. 2019), run at the same horizontal resolution as the atmosphere. CESM2 also features a new interactive ice sheet component (Muntjewerf et al. 2020a, b), the Community Ice Sheet Model version 2.1 (Lipscomb et al. 2019), at a default 4 km resolution. In this study, the ice sheet evolution is turned off (fixed topography), so the ice sheet model is purely diagnostic. CESM2 successfully simulates present-day GrIS SMB (van Kampenhout et al. 2020), and reproduces the SMB response to global warming as simulated by high-resolution regional climate models (van Kampenhout et al. 2020). It has also been applied to several studies projecting the SMB response to global warming for a fixed present-day (Sellevold and Vizcaino 2020) and evolving (Muntjewerf et al. 2020a, b) topography.

### Surface mass balance calculation

For the calculation of GrIS SMB, CESM2 uses an elevation class (EC) scheme (Sellevold et al. 2019). CLM5 carries out this calculation at every grid cell over Greenland containing any glacier cover. The EC method downscales the near-surface temperature with a lapse rate of 6 K km$^{-1}$, the incoming longwave radiation with 32 W m$^{-2}$ km$^{-1}$, and specific humidity assuming constant relative humidity, using ten elevation bins. For the calculation of surface melt, the surface energy balance [W m$^{-2}$] is calculated at every EC as

1 $$\begin{aligned} M = SW_{in}(1-\alpha ) + LW_{in}-\epsilon \sigma T_{sfc}^{4} + SHF + LHF + GHF, \end{aligned}$$

where M is the melt energy, SW$_{in}$ is the incoming shortwave, $\alpha$ is the surface albedo, LW$_{in}$ is the incoming longwave, $\epsilon$ the surface emissivity, $\sigma$ the Stefan–Boltzmann constant, and T$_{sfc}$ the surface temperature. SHF is the sensible heat flux, LHF is the latent heat flux, and GHF is the ground heat flux. The condition for M to be positive, i.e., for surface melt to occur, is that the surface temperature is at the melting point.

The snow albedo is explicitly simulated by the Snow, Ice, and Aerosol Radiative (SNICAR) model (Flanner and Zender 2006). The albedo of snow depends on the solar zenith angle, the concentration of deposited aerosols, and dry and liquid snow aging processes. The effective radius of freshly fallen snow is based on the near-surface temperature (van Kampenhout et al. 2020). When all snow has melted over a glaciated surface, bare ice is exposed. The bare ice albedo is fixed at 0.5 for visible radiation and 0.3 for near-infrared radiation.

At every EC, the SMB [Gt year$^{-1}$] is calculated as

2 $$\begin{aligned} SMB = SNOW + REFRZ - MELT - SUBL, \end{aligned}$$

where SNOW is the snowfall, REFRZ is the refreezing of rainfall ﻿or melt, MELT is the surface melt, and SUBL is the sublimation (deposition if negative). Phase partitioning of rain and snowfall occurs at each EC based on near-surface temperature. At temperatures lower than $-2\,^{\circ }$C, precipitation falls exclusively as snow, while at temperatures higher than 0 $^{\circ }$C, precipitation falls exclusively as rain. In between this range, it appears as mixed-phase precipitation.

**Fig. 1:**
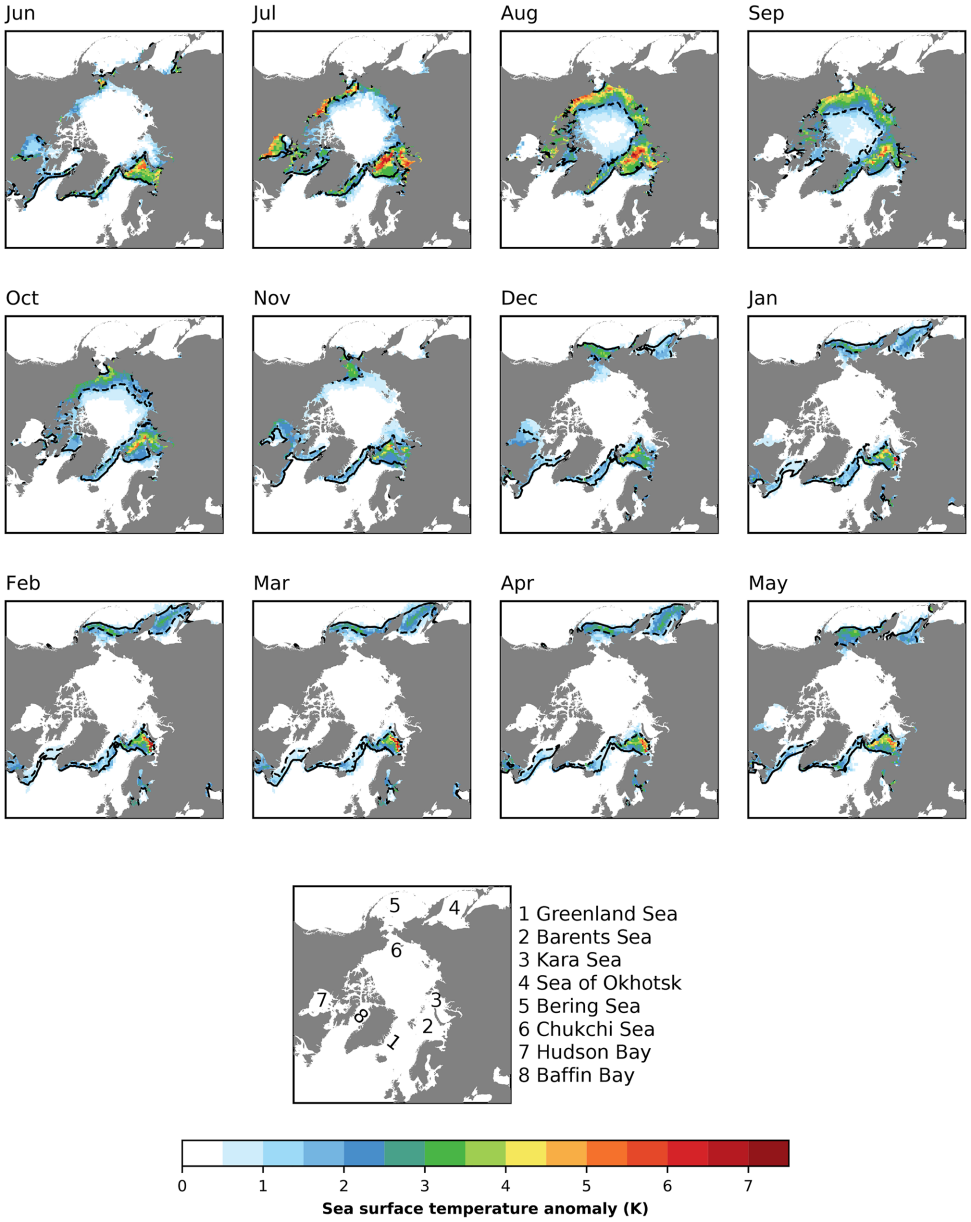
Sea-ice extent and sea surface temperature differences between future (FUT) and control (CTRL) experiments for the months of the year. The solid black line corresponds to the CTRL sea ice edge (>0.15 SIC), while the black dashed line corresponds to the FUT sea ice edge. The color corresponds to the difference in prescribed SST between FUT and CTRL. The lowest panel shows the location of places we mention

### Simulations

The simulations analyzed here are contributions to the Polar Amplification Model Intercomparison Project (PAMIP; Smith et al. 2019). Two experimental setups are used, both starting in April 2000 and running through May 2001. The first two months are discarded as spinup, leaving a full year for analysis. Each of the two experiments consists of 100 1-year simulations, each with slightly different (bit perturbed) initial atmospheric conditions. The difference between the two experiments is only in the prescribed sea ice and within-Arctic SSTs, one corresponding to pre-industrial conditions (CTRL; Danabasoglu 2019a) (experiment number 1.5; Smith et al. 2019) and the other to a 2 $^{\circ }$C warmer climate (FUT; Danabasoglu 2019b) (experiment number 1.6; Smith et al. 2019) as illustrated in Fig. 1.

In the simulations we analyze, the boundary forcing consists of varying sea ice concentrations and SSTs with one-month frequency. The monthly varying sea ice concentrations and SSTs were obtained from the historical and RCP8.5 scenario simulations from the CMIP5 (Taylor et al. 2012). Three distinct periods are defined: Pre-industrial, present-day, and future, with global mean temperatures of 13.7 $^{\circ }$C, 14.2 $^{\circ }$C, and 15.7 $^{\circ }$C, respectively. For each CMIP5 model, the 30-year running mean global mean temperature is calculated. When this global mean temperature matches those defined above, a 30 year average of SIC and SST is taken to represent the period. At each grid point, linear regression between present-day values and pre-industrial (or future) values of SIC and SST across the 30-year averages from each model are computed. Then, the required pre-industrial (or future) estimate is taken as the point where this regression relationship intersects the observed (1979–2008 climatology from the Hadley Centre Sea Ice and Sea Surface Temperature dataset; Rayner et al. 2003) values to constrain the estimates of SIC and SST. In the linear regression to obtain SIC and SST, quartile regression (Waldmann 2018) is used instead of the more common least square regression to reduce the influence of outliers. For the pre-industrial (future), the upper (lower) quartile of the regression is used to give higher weight to models with more (less) sea ice and colder (warmer) SST’s. Following the method of Screen et al. (2013b), in any grid cell where the pre-industrial or future SIC deviates with more than 10% from the present-day value, SSTs derived using the method described above are prescribed.

### Analysis

To assess the response to Arctic sea ice loss and increased SSTs, we make use of some specific circulation metrics.

To identify individual cyclones, we use a modified version of the method presented in Zhang et al. (2004). The method applies these steps on 6 hourly averaged sea level pressure (SLP) data:

1. Remove SLP values where the surface elevation is higher or equal to 1000 m.
2. Any grid point with SLP lower than its eight surrounding neighbors is considered a cyclone candidate.
3. The minimum absolute SLP gradient between the cyclone candidate and its eight surrounding grid points is required to be 1.5 $\times$ 10$^{-6}$ hPa m$^{-1}$. The SLP values at the eight surrounding grid points are representative of the spatial average using their nine adjacent grid points.
4. The minimum SLP gradient between the four surrounding points of the cyclone candidate and their outside adjacent grid points must be negative inward.
5. We add a radius of 600 km to each cyclone center.
6. The daily average of cyclones is calculated, to obtain the fraction of a day when a cyclone influences a grid point.

We expect that different cyclone detection algorithms give different results. However, the cyclone climatology produced with the method presented here (Fig. S1) compares well with those of other methods (Neu et al. 2013).

To identify dynamic blocking events, we use the modified two-dimensional method described by Kennedy et al. (2016). We take the 5-day running mean of daily averaged 500 hPa geopotential heights (Z$_{500}$) to enforce a 5-day criterion on the duration of a blocking event. At every grid point within 35$^{\circ }$ N and 80$^{\circ }$ N, we calculate the northern (G$_N$) and southern (G$_S$) gradients through the formula

3 $$\begin{aligned} G_S = \frac{Z_{500}(\phi _0) - Z_{500}(\phi _S)}{\phi _0 - \phi _S}, \quad G_N = \frac{Z_{500}(\phi _N) - Z_{500}(\phi _0)}{\phi _N - \phi _0}, \end{aligned}$$

where $\phi _0$ corresponds to the latitude of the grid cell, $\phi _N$ = $\phi _0$ + 10$^{\circ }$ N, and $\phi _S$ = $\phi _0 - 10 \,^{\circ }$ N. Whenever G$_S$ > 0 and G$_N$ $< \,-$10 m degree$^{-1}$, we consider the grid cell blocked. The difference of our method compared to the original method, is that we calculate gradients with a distance of 10$^{\circ }$ N rather than 15$^{\circ }$N. This allows us to extend to 80$^{\circ }$ N, while the original method can calculate up to 75$^{\circ }$ N. However, the two methods give similar results within the overlapping area (not shown).

## Results

### Large-scale climate response

In this section, we explore the Arctic response in CESM2 to the monthly varying SIC and SST perturbations shown in Fig. 1. Sea ice is reduced every month of the year and is accompanied by a co-located increase in SST. The most widespread loss of sea ice occurs in summer and late fall. In the other months, the largest sea ice losses occur in the Barents Sea, the Greenland Sea, the Bering Sea, and the Sea of Okhotsk. In the seas surrounding Greenland, there is a year-round loss of sea ice. The sea ice reductions in the Arctic (> 60$^{\circ }$) are 3.6 $\times$ 10$^6$ km$^2$ (19.0%) and 6.4 $\times$ 10$^6$ km$^2$ (48.4%) for winter and summer, respectively. The corresponding SST increases are 0.4 K and 1.4 K.

**Fig. 2:**
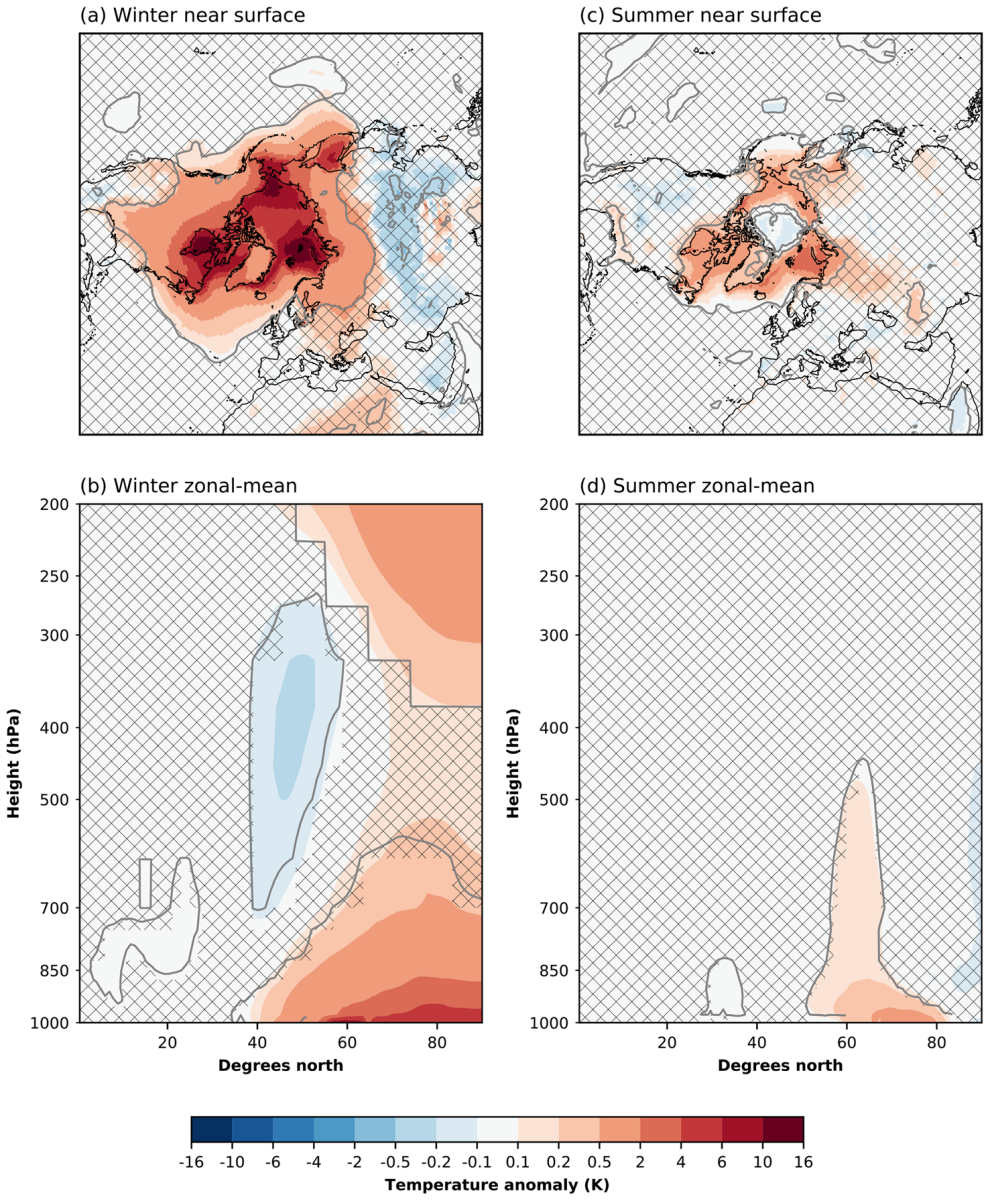
Simulated Arctic amplification as a response to the forcing. **a** Winter (DJF) near surface [K], **b** winter zonal-mean [K], **c** summer (JJA) near surface [K], and **d** summer zonal-mean [K] temperature anomalies. The differences are for the ensemble means over all 100 members of each experiment. Patched areas enclosed by grey contours indicate non-significant response at 95 % level, according to a student’s t-test

In response to this forcing, the Arctic experiences atmospheric warming, mostly in winter but also in summer (Fig. 2a, c). In winter, there is widespread warming over both the Arctic ocean and Arctic land (Fig. 2a). Three local warming maxima can be found in the Barents–Kara Seas, the Hudson Bay, and the Chukchi Sea. The warming imprint is evident over the entire Greenland ice sheet, with the strongest warming occurring in the South. The Arctic surface warming extends to a depth of $\sim$ 600 hPa (Fig. 2b). There is also warming occurring in the upper troposphere in response to the sea ice and SST forcing. There is some mid-tropospheric cooling around 40$^{\circ }$ N, associated with a weak (not significant) surface signature in central Asia.

During summer, the warming is primarily confined to the ocean in a latitudinal band of 50$^{\circ }$ N–80$^{\circ }$ N (Fig. 2c). Also, the southern part of Greenland warms. Central parts of the Arctic Ocean show a small (0.1 K) yet significant cooling. In the zonal-mean, the Arctic surface warms, and this warming extends into the troposphere (up to $\sim$ 450 hPa; Fig. 2d).

**Fig. 3:**
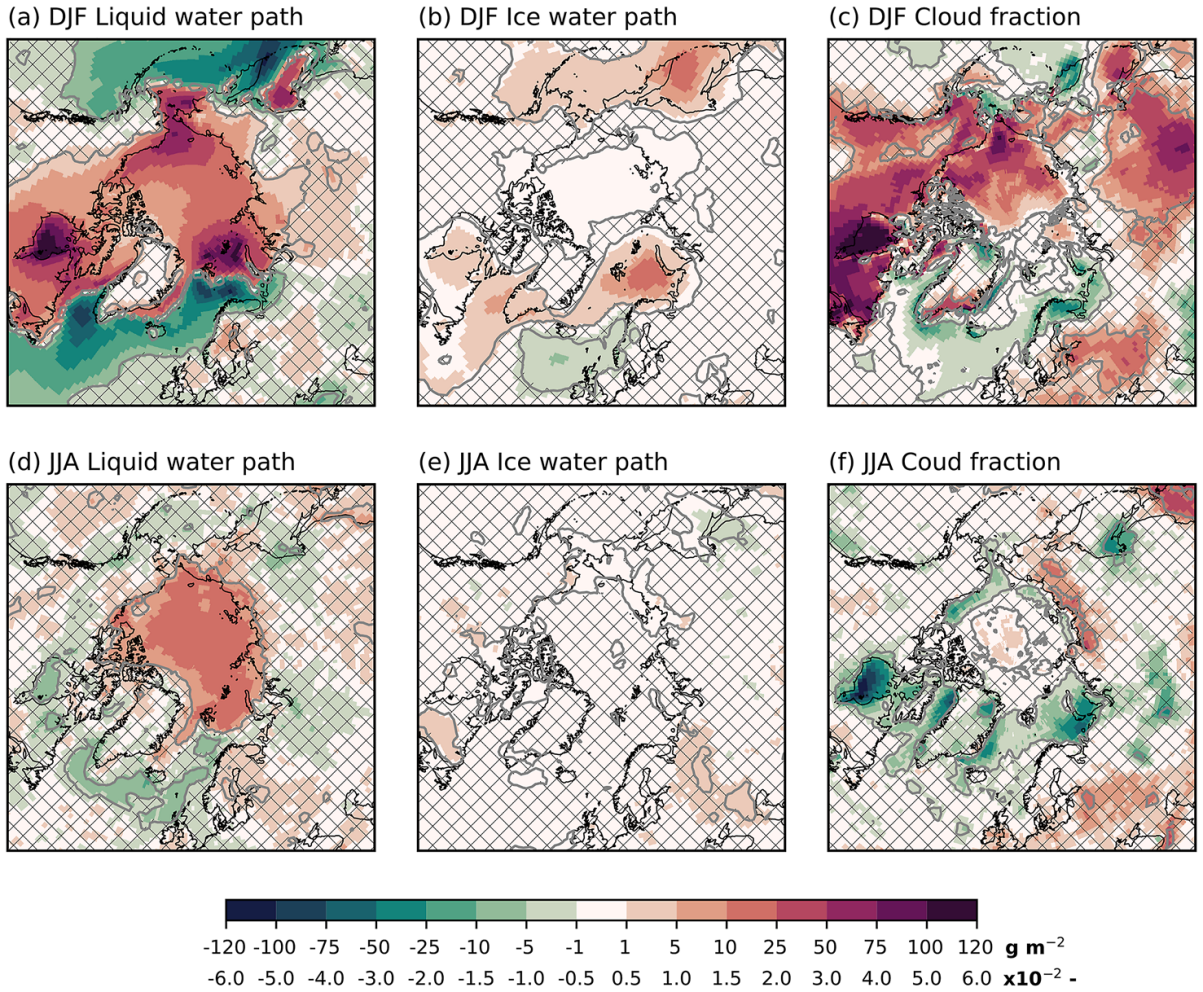
Arctic cloud anomalies from sea-ice reduction (FUT minus CTRL). **a** + **d** Liquid water path [g m$^{-2}$], **b** + **e** ice water path [g m$^{-2}$], and **c** + **f** cloud fraction [−]. The upper row corresponds to winter (DJF), the lower row to summer (JJA). Patched areas enclosed by grey contours indicate non-significant response at 95% level, according to a student’s t-test

A more cloudy Arctic accompanies the warming. In winter, there is a pan-Arctic increase in cloud liquid water (Fig. 3a) with maxima located in the regions of largest sea-ice reductions. Co-located with these maxima is also an increase in cloud ice water (Fig. 3b). Clouds also become more frequent in the Arctic, except for over the Barents Sea, where the model simulates reduced cloud fraction (Fig. 3c). In the Pacific and the North Atlantic, clouds become less frequent and contain less liquid water. On the other hand, the cloud ice water increases.

The changes in summer clouds are smaller than in winter. In the central Arctic, the cloud liquid water increases, while there are non-significant differences in the cloud ice water (Fig. 3d, e). Areas experiencing sea-ice loss have a reduced cloud fraction, while the area in the central Arctic with no changes in the sea-ice has an increase in cloud fraction (Fig. 3f).

**Fig. 4:**
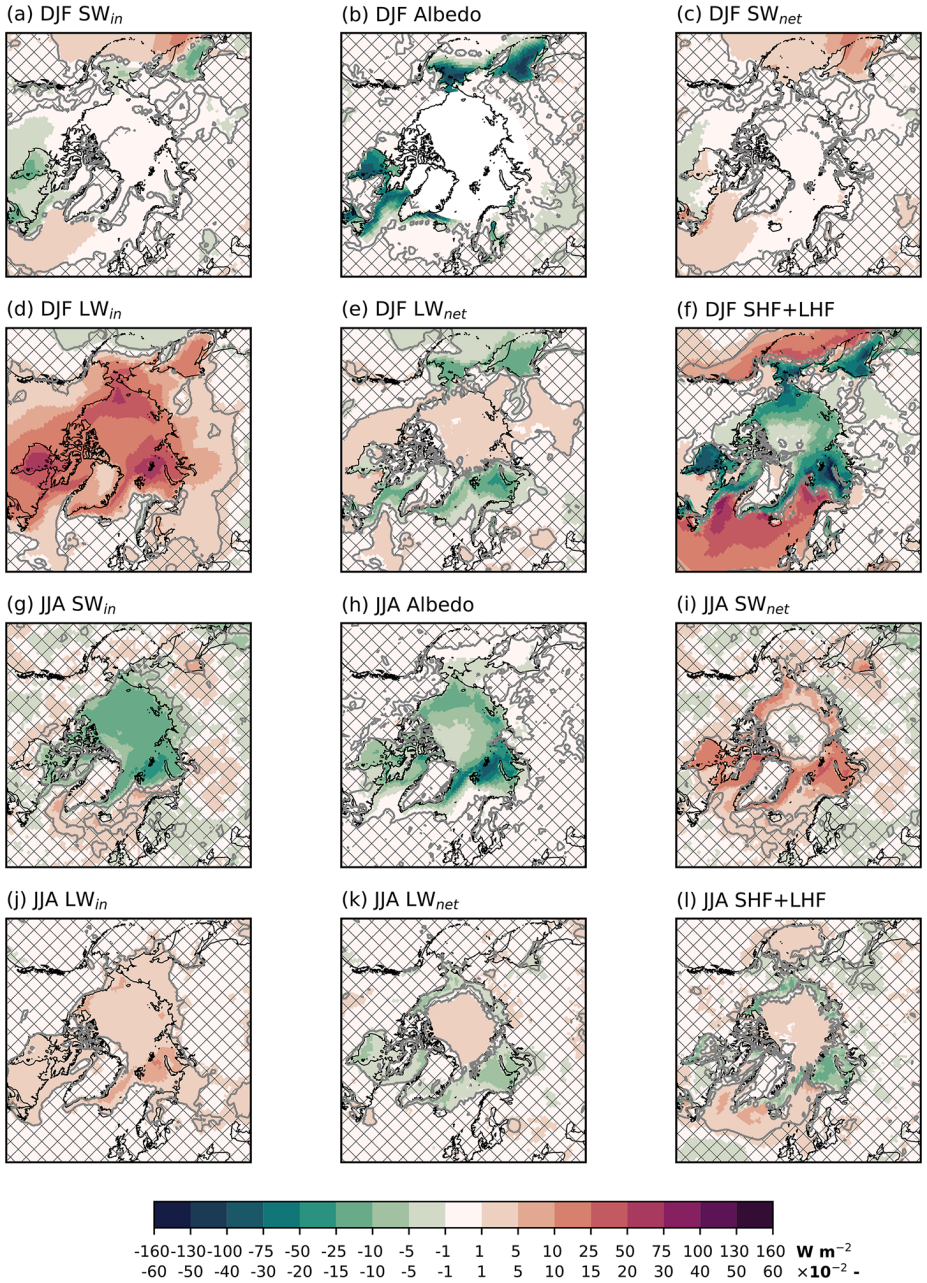
Arctic surface energy components’ anomalies in FUT with respect to CTRL. **a** + **g** SW$_{in}$ [W m$^{-2}$], **b**+ **h** albedo [−], **c**+ **i** SW$_{net}$ [W m$^{-2}$], **d** + **j** LW$_{in}$ [W m$^{-2}$], **e**+ **k** LW$_{net}$ [W m$^{-2}$], and **f** + **l** SHF+LHF [W m$^{-2}$]. The convention for SHF+LHF is that positive means increased energy transfer to the surface. The upper two rows contains winter (DJF) averaged quantities, the lower two rows summer (JJA) averaged. Patched areas enclosed by grey contours indicate non-significant response at 95 % level, according to a student’s t-test

Figure 4 shows different components of the Arctic surface energy budget. In winter, there is a decrease in SW$_{in}$ over the Hudson Bay and the Sea of Okhotsk (Fig. 4a), both due to increased cloudiness and a lower surface albedo (Fig. 4b, Fig. S2). The decrease in SW$_{in}$ is more than compensated for by a decrease in albedo, leading to increased SW$_{net}$ (Fig. 4c). There is a pan-Arctic increase in LW$_{in}$ (Fig. 4d) caused by increased atmospheric re-emittance of LW radiation and more clouds as the atmosphere warms and moistens. The patterns follow the near-surface air temperature response pattern closely (Fig. 2a). However, in these areas with higher near-surface air temperature, the LW$_{out}$ increases more than the LW$_{in}$ due to high warming of the surface, leading to decreased LW$_{net}$ (Fig. 4e). In the central Arctic Ocean and over Arctic landmasses in Siberia and Canada, the LW$_{net}$ increases as a result of increasing LW$_{in}$. In these areas, the snow or ice-covered surfaces does not warm enough to compensate for the increased LW$_{in}$. Where sea ice is lost in FUT, SHF+LHF decreases substantially (i.e., more transfer of heat from the surface to the atmosphere) in the Barents-Kara Seas, Hudson Bay, the Chukchi Sea, and in the Sea of Okhotsk (Fig. 4f). The SHF+LHF decrease is due to the prescribed strong SST warming and sea ice loss at the boundary of a relatively cold atmosphere. Where sea ice is perturbed, more heat and moisture enter the atmosphere, which winds advect over the North Atlantic and the Pacific Ocean, where there is no change in surface conditions. This leads to an increase in SHF+LHF in these areas. The increase represents less heat and moisture transfer from the surface to the atmosphere.

In summer, large parts of the Arctic Ocean experience more cloudiness and reduced albedo, leading to a reduction in SW$_{in}$ (Fig. 4g, h). As in winter, in areas with sea ice loss, the SW$_{net}$ increases (Fig. 4i) despite the reduction in SW$_{in}$ due to reduction in surface albedo. This effect is more prominent in summer as the background solar insolation is higher in summer. Due to the increased cloud cover and higher atmospheric temperatures, the LW$_{in}$ increases. This increase is much smaller in summer than in winter, as the summer’s atmospheric temperature response is smaller. The patterns of LW$_{net}$ largely correspond to those of winter (Fig. 4k). The Arctic SHF+LHF is much smaller in summer than in winter (Fig. 4l), due mainly to the reduced temperature contrast between the atmosphere and the ocean. There is an increase in SHF+LHF in the central Arctic, so the atmosphere transfers heat to the surface (Fig. 2c).

### GrIS surface mass balance response

**Table 1:** GrIS integrated mass components in winter (DJF) and summer (JJA), all in Gt year$^{-1}$

Simulation	SMB	Precipitation	Melt
*DJF*			
CTRL	177 ± 34	164 ± 33	0 ± 0
FUT	**200 ± 32**	**186 ± 32**	0 ± 0
*JJA*			
CTRL	$-$123 ± 64	231 ± 36	454 ± 75
FUT	**− 156 ± 63**	226 ± 36	**490 ± 77**

The standard deviation is given by ±, and bold values indicate a significant response at the 95% level according to a student’s t-test

**Fig. 5:**
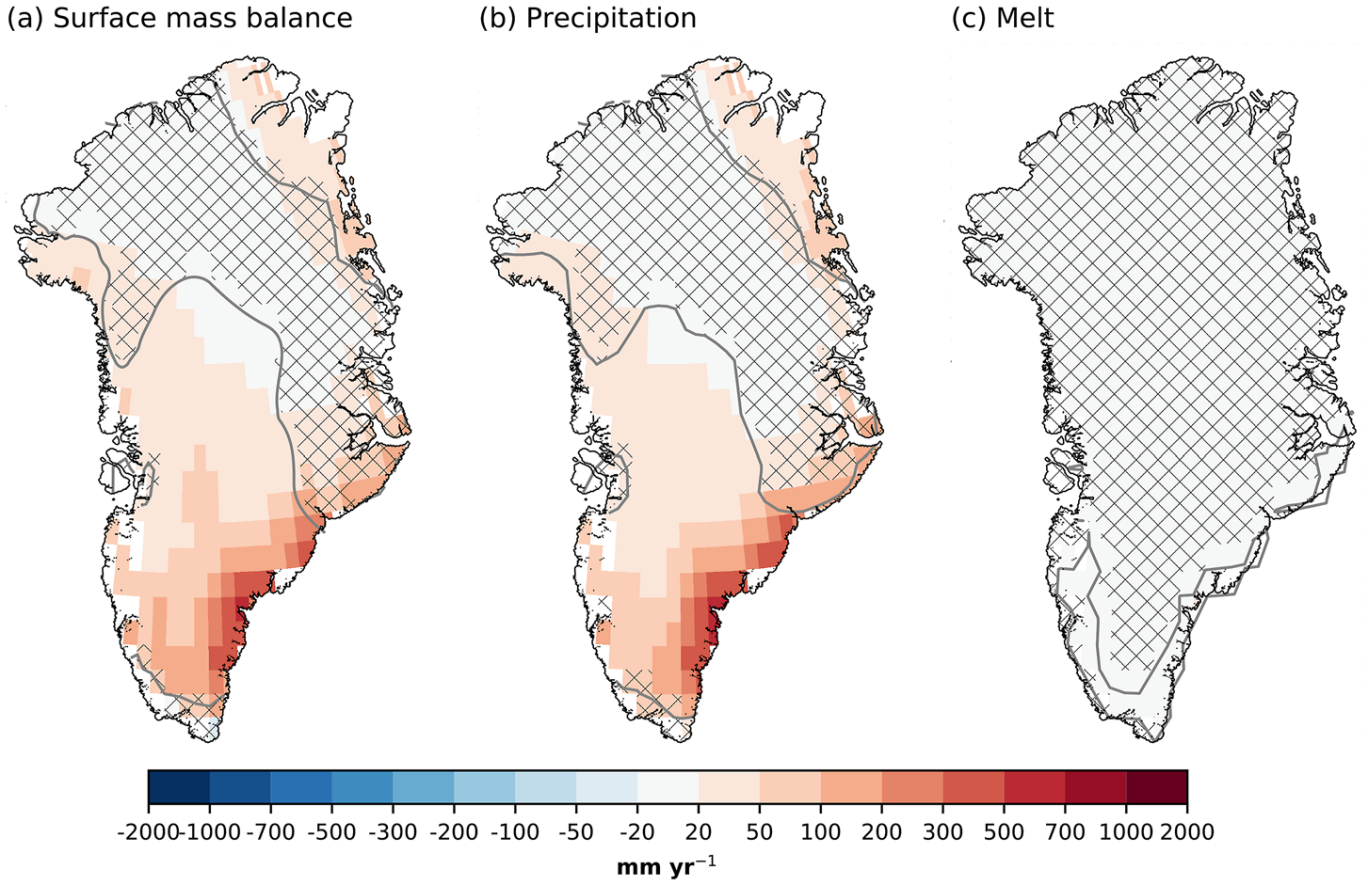
GrIS mass anomalies in FUT with respect to CTRL during winter (DJF). **a** Surface mass balance, **b** precipitation, and **c** melt, all in mm year$^{-1}$. Patched areas enclosed by grey contours indicate non-significant response at 95 % level, according to a student’s t-test. Only values corresponding to the glaciated part of the grid cell are shown

Sea ice loss and ocean warming increase the winter SMB over the GrIS by 23 ± 33 Gt year$^{-1}$ (Table 1). SMB increases on 55% of the ice sheet. There is no significant change in a broad region ranging from the northwest across the high-elevation interior to the east (Fig. 5a). The largest increase in SMB is in the high accumulation area in the southeast. In relative terms, the increase in the northwest and northeast is as large. The main cause of these SMB increases is a 22 ± 33 Gt year$^{-1}$ increase in precipitation (Table 1, Fig. 5b). Despite ice-sheet-wide winter warming over the GrIS, melt does not increase (Fig. 5c) because temperatures remain below freezing.

**Fig. 6:**
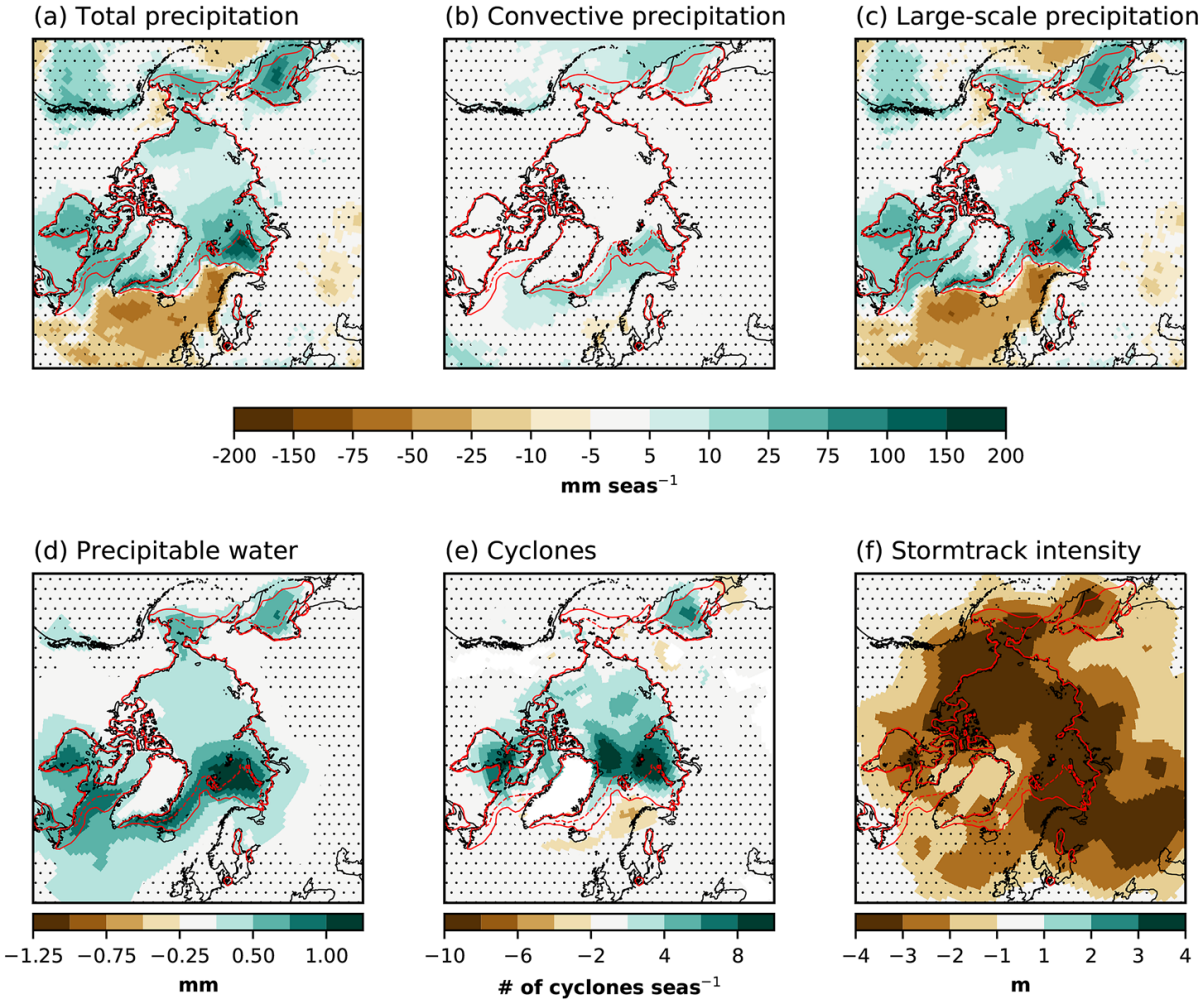
Precipitation and storm responses in FUT with respect to CTRL in winter. **a** Total precipitation [mm seas$^{-1}$], **b** convective precipitation [mm seas$^{-1}$], **c** large-scale precipitation [mm seas$^{-1}$], **d** column-integrated precipitable water [mm], **e** number of cyclones per season, and **f** storm track intensity [m]. The storm track intensity is calculated as the standard deviation of the 2–6 days bandpass filtered Z$_{500}$. The solid (dotted) red line is the sea ice extent from CTRL (FUT). Dots indicate non-significant responses at the 95% level

To understand the increased winter precipitation over Greenland, we explore the atmospheric dynamics in the Arctic and the North Atlantic. The total amount of precipitation increases in the Arctic (Fig. 6a), where the largest increases are in areas with sea ice loss. A smaller part of the total precipitation increase can be attributed to increased convection (Fig. 6b). Increased convection mainly occurs in areas with sea ice loss. Large-scale precipitation is the main contributor to Arctic precipitation increase (Fig. 6c), and the anomalies highly resemble the total precipitation anomalies. The phase of precipitation does not change significantly due to the cold Arctic winter climate. The precipitable water increases everywhere in the Arctic (Fig. 6d). The largest increases in precipitable water are consistent with positive SHF+LHF anomalies (Fig. 4e), as these are the locations where more water enters the atmosphere in FUT with respect to CTRL. Also, the warmer atmosphere is capable of holding more water from both local and remote sources. Further, this moisture is advected to the central Arctic and southward to, e.g., the North Atlantic. The frequency of cyclones increases (Fig. 6e) in the Arctic, likely due to enhanced baroclinicity through destabilization of the atmosphere. The latter is in response to higher SHF+LHF caused by a stronger surface-to-atmosphere temperature gradient. Together with relatively much increased atmospheric moisture, precipitation increases even though the storm intensity is reduced (Fig. 6f).

An interesting feature of the large-scale precipitation response is the contrast between the Arctic and the North Atlantic, with reduced precipitation in the North Atlantic. There are fewer cyclones (Fig. 6e) and weaker storm tracks (Fig. 6f), which is consistent with reduced large-scale precipitation, despite the increase in atmospheric moisture. A plausible explanation for the storm response is that ocean temperatures in the North Atlantic are identical in the runs, while the atmosphere experiences increases in heat and moisture (Figs. 2a, 6d) in the North Atlantic. This reduces the climatological ocean-to-atmosphere SHF+LHF (Fig. 4e) and may act to stabilize the atmosphere (Fig. S3c). The stabilization, together with a weaker equator-to-pole SST gradient, reduces the baroclinicity in the North Atlantic (Fig. 6e).

**Fig. 7:**
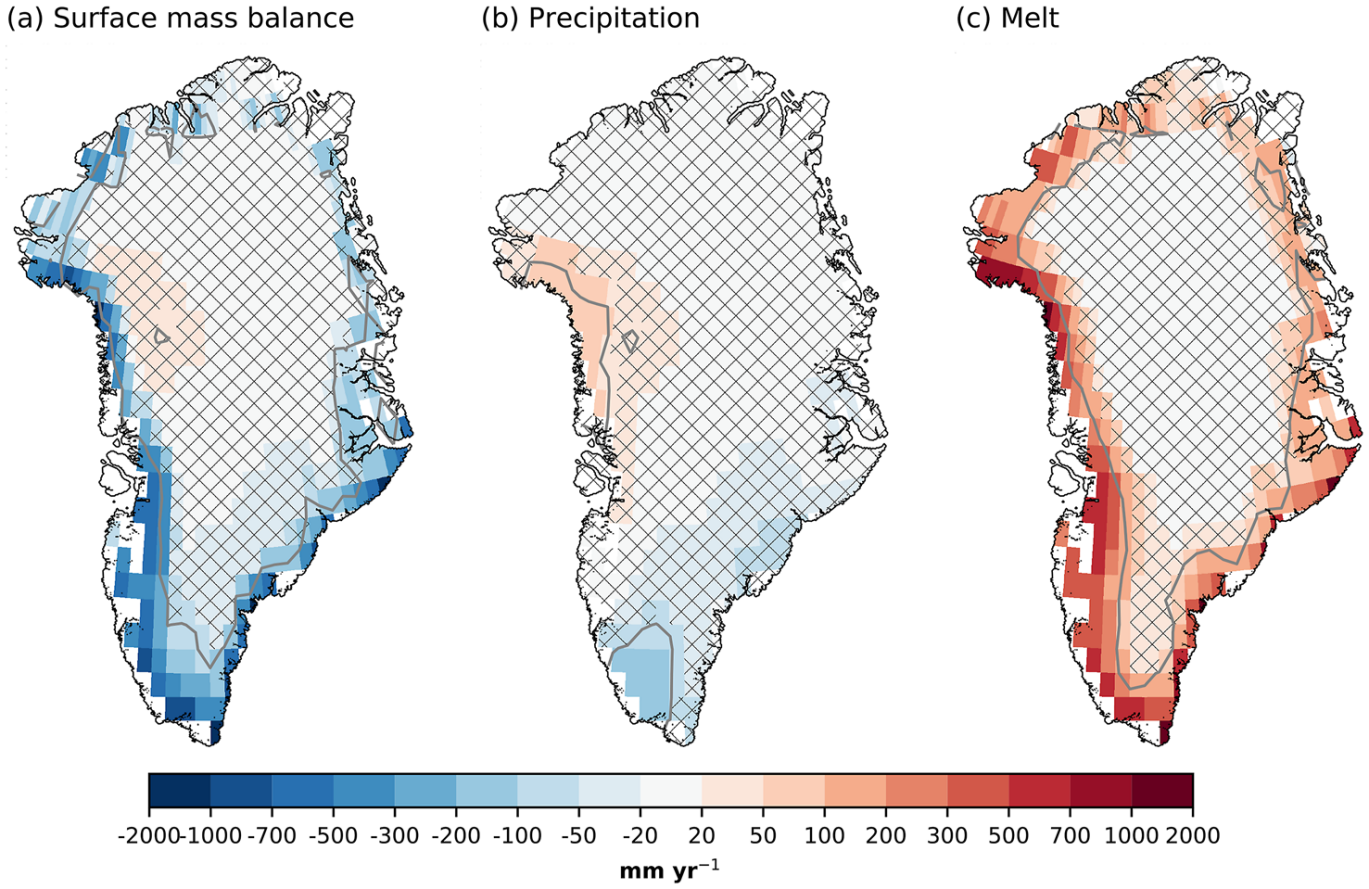
Same as Fig. 5, but for summer (JJA)

SMB decreases along with coastal, low elevation areas of the GrIS in summer (Fig. 7a). The total SMB decrease is 33 ± 64 Gt year$^{-1}$ (Table 1). The main component of this decreased SMB is melt increase (Fig. 7f), which increases by 36 ± 76 Gt year$^{-1}$ (Table 1). The summer precipitation response shows a dipole structure, with increased precipitation in the high accumulation area in the northwest and decreased precipitation in the South. These two precipitation anomalies approximately cancel each other in the total mass budget, leading to a small, non-significant decrease in the integrated summer SMB.

**Fig. 8:**
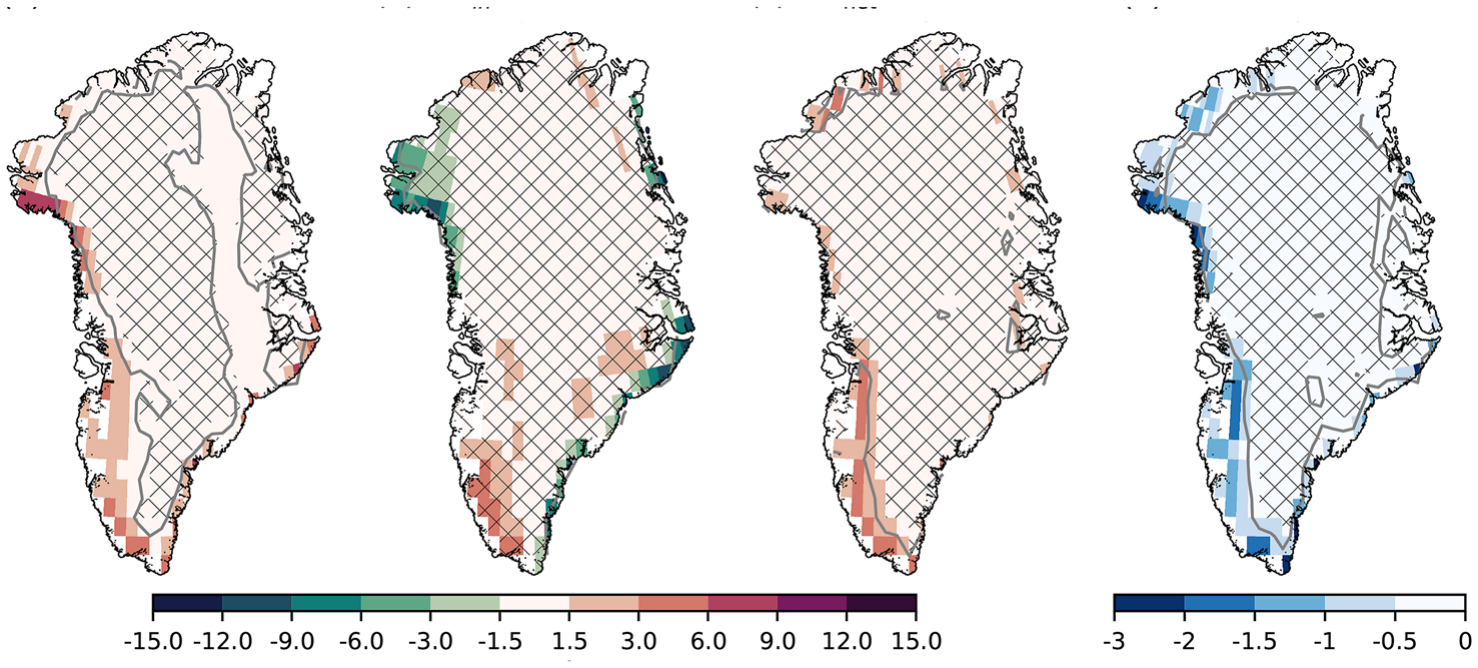
Summer GrIS surface energy balance response to the sea ice forcing. **a** SHF+LHF [W m$^{-2}$], **b** SW$_{in}$ [W m$^{-2}$], **c** SW$_{net}$ [W m$^{-2}$], and **d** albedo [−]. Patched areas enclosed by grey contours indicate non-significant response at 95 % level, according to a student’s t-test. Only values corresponding to the glaciated part of the grid cell are shown

**Fig. 9:**
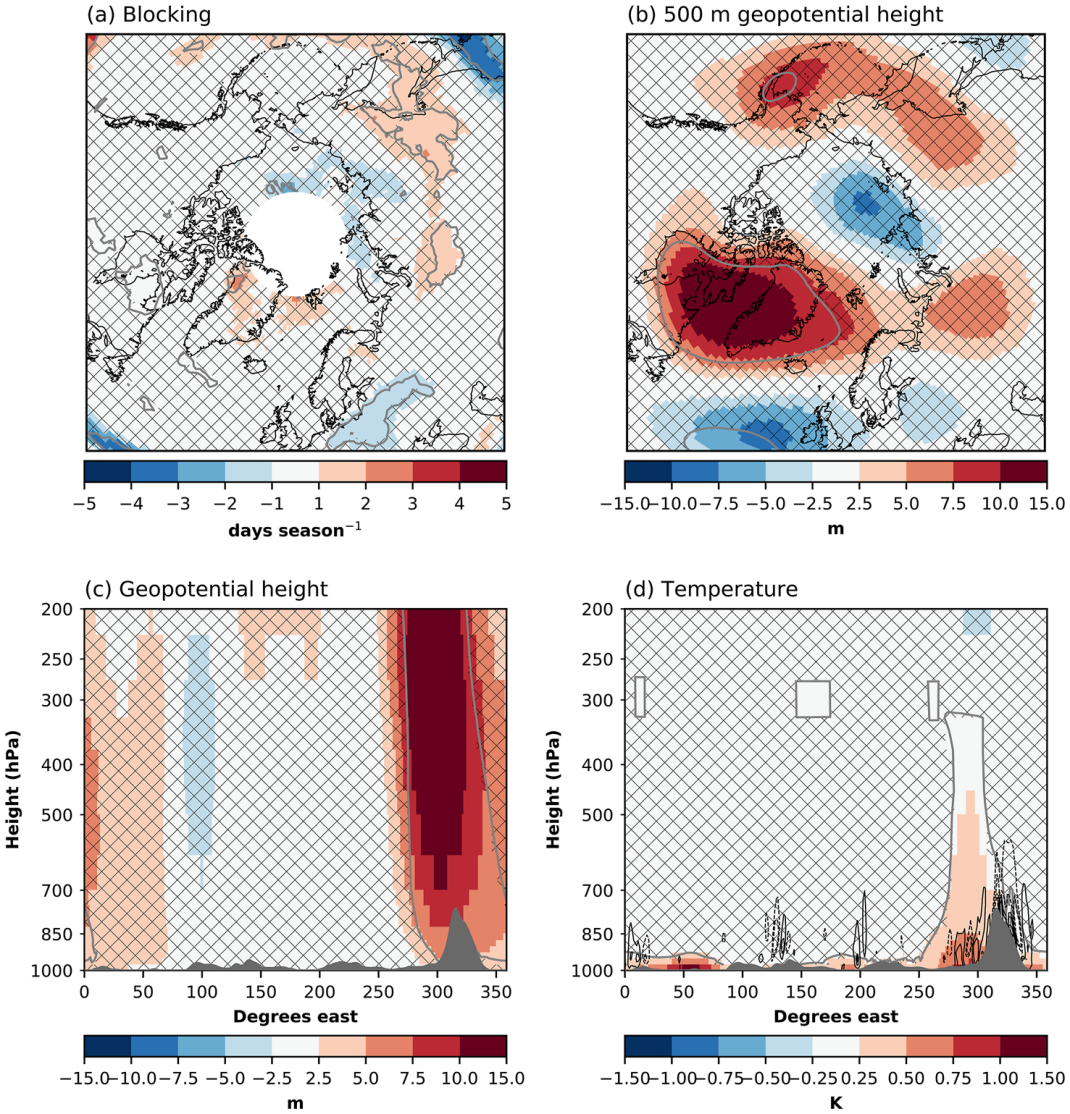
Summer atmospheric circulation responses to sea ice forcing. **a** Blocking days [days seas$^{-1}$], **b** 500 hPa geopotential height [m], **c** meridional-mean geopotential height [m], and **d** meridional-mean temperature [m]. For **c** and **d**, the meridional-mean is taken between 60$^{\circ }$ N and 80$^{\circ }$ N. The contours in **d** represent vertical velocities, scaled by the horizontal wind speed. The contour levels are 0.25, 0.5, 0.75, 1, and 1.5, and are symmetric around zero. Solid lines show positive values, while dashed lines show negative values. Patched areas enclosed by grey contour lines indicate non-significant differences at the 95% level

The summer melt increase, leading to a lower summer SMB, can be explained through changes in the surface energy balance. Along the margins of GrIS, SHF+LHF increases (i.e., more turbulent energy transfer from the atmosphere to the ice sheet surface). Increased SHF+LHF (Fig. 8a) occurs as the atmosphere warms (Fig. 2a) and moistens. The largest responses are found in the western part of the GrIS, which two factors can explain. First, this is an area where the ice sheet experiences the highest melt during the summer, so the surface temperature is at 0 $^{\circ }$C for a long time in the summer, also in the absence of sea ice forcing. When the surface is at 0 $^{\circ }$C, any additional atmospheric warming increases the surface-to-atmosphere temperature contrast leading to higher SHF+LHF, as opposed to when the surface also warms. Second, Baffin Bay is one of the areas warming the most during summer. Anomalous winds (wind speeds increase anticyclonically parallel to the positive height contours) cause the positive south-to-north SHF+LHF anomaly gradient in the West, with faster winds in the north and slower winds in the South (Fig. 9b,c). Despite the SW$_{in}$ not showing a statistically significant response (Fig. 8b) to the sea ice forcing, the SW$_{net}$ increases along the margins (Fig. 8c). The increased absorption of SW energy is due to a lower albedo (Fig. 8d). The lowered albedo is due to earlier melt and a 3.5% increase in the summer melt extent, and a 0.6% increase in the bare-ice area. The higher SHF+LHF likely provides the energy for the initial melt, triggering the melt-albedo feedback (Box et al. 2012).

Summer sea ice loss influences the atmospheric circulation over the GrIS. A robust, highly localized increase in blocking events can be detected over the northwestern GrIS (Fig. 9a). There is an increase in 2–3 days ($\sim$ 25%) with blocked atmospheric circulation in this region. Increased blocking is related to melt by sustained warm air advection over the ice sheet. Further, the increased blocking in this region is consistent with the higher SHF+LHF (Fig. 8a). This increase in blocking is accompanied by a larger-scale increase in Z$_{500}$ centralized over the Baffin Bay (Fig. 9b). This anomalous circulation pattern increases wind speeds in the northwestern part of the ice sheet and slows down winds in the southwestern part. This explains the dipole precipitation pattern (Fig. 7b), as the winds affect the amount of moisture transfer over the ice sheet. Further, this circulation anomaly is similar to the circulation anomaly associated with the Greenland blocking index (Davini et al. 2012; Hanna et al. 2015, 2018). However, the present-day GBI-related anomaly is approximately 6 $\times$ stronger than the circulation anomaly found here (Hanna et al. 2016). Still, this indicates that sea-ice loss modulates the strength of the GBI. The geopotential height anomaly is deep, with an equivalent barotropic structure (Fig. 9c). This deep anomaly occurs only over the Baffin Bay/Greenland, although the strongest surface forcing is not located there (Fig. 1a–c).

The Baffin Bay/Greenland region is also the region where the temperature response is deepest, extending from the surface and up to $\sim$ 300 hPa (Fig. 9d). Deeper heating of the atmosphere has been related to a stronger upper-level geopotential height response (Sellevold et al. 2016). We hypothesize that this deep atmospheric heating is due to the strong vertical winds at the coast of the GrIS. The free-atmosphere wind flow in the polar/extratropical northern hemisphere is predominantly westerly. The GrIS acts as a barrier to this flow, forcing vertical motion, and possibly triggers gravity waves (Doyle et al. 2005; Limpasuvan et al. 2007; Harden and Renfrew 2012), enhancing the turbulent mixing of air (Vosper et al. 2018) (Fig. 9d). This way, the high elevation of the GrIS together with sea ice loss generate an anomalous circulation pattern that increases the ice sheet’s surface melt.

## Discussion

We investigated the impact of reduced Arctic sea ice on GrIS SMB by forcing CESM2 with pre-industrial and future (corresponding to +2 $^{\circ }$C global mean temperature) monthly varying SIC and SST. CESM2 is suited to address this question as it is one of the few global models including a realistic (van Kampenhout et al. 2020), interactive calculation of the SMB in the land component, with advanced snow and firn physics (van Kampenhout et al. 2017) and downscaling via elevation classes (Sellevold et al. 2019). We found an ice-sheet-wide significant increase in precipitation during the winter months. The model simulated future summer increases in melt around the entire margin, with the strongest responses in the West of the ice sheet.

The results presented here rely on idealized SIC and SST perturbations to isolate the impact of sea ice loss on the GrIS. However, this experimental setup does not capture some indirect effects. For example, sea ice loss may cause warming over lower latitude oceans (Blackport and Kushner 2017) altering the North Atlantic responses reported here. In turn, these changes in North Atlantic responses may affect the simulated response of the GrIS.

In our study, the Arctic becomes warmer and more humid in response to sea ice reductions. In winter, the driving processes are increased ocean-to-atmosphere turbulent heat loss and more incoming (downward) longwave radiation. In summer, increased net solar radiation through reduced albedo where sea ice transitions to open ocean adds to the warming. These Arctic responses are robust among climate simulations with a similar setup (e.g., Deser et al. 2010; Screen et al. 2013b; Peings and Magnusdottir 2014). In our results, turbulent heat gain south of the sea ice edge is likely overestimated due to the lack of ocean coupling. Still, it is present in coupled simulations of global warming (Sellevold and Vizcaino 2020) as the atmosphere warms faster than the ocean.

Precipitation over the GrIS increases in winter. This response was also identified by Noël et al. (2014). However, the response they found was confined to the southeast, while here, we found widespread precipitation increase over the GrIS. One possible explanation for this discrepancy could be the difference in model resolution, as they use a regional climate model. The lower resolution here, with associated smoothed topography over the GrIS, may allow for moisture to travel further into the ice sheet.

McIlhattan et al. (2020) shows that Arctic precipitation frequency from liquid-containing clouds has increased in CESM2 with respect to CESM1 during all months of the year, and the annual mean is very high (0.642) in CESM2 compared to observations (0.129). However, the total amount of precipitation in CESM2 has not increased in comparison to CESM1. As the main focus in this study is the integrated amount of precipitation over the GrIS, we deem the integrated amount of precipitation reliable. While we cannot assess the impact of higher frequency precipitation on, e.g., summer albedo, and consequently, ice sheet melt with the simulations analyzed here, this could be addressed in a follow-up study.

Enhanced summer melting at low elevation areas of the GrIS, as found here, is also a robust response to reduced Arctic sea ice cover (Rennermalm et al. 2009; Noël et al. 2014; Stroeve et al. 2017; Pedersen and Christensen 2019). Liu et al. (2016) argue that the primary mechanism for the increased surface melt of the GrIS is through increased LW$_{in}$ by atmospheric warming caused by sea ice loss. Here we find that LW$_{in}$ only significantly increases for a limited area in the northwest due to the limited warming of the GrIS in the summer. We find the primary mechanisms for increased melt to be a triggering of the albedo-melt feedback by increased SHF+LHF. The effect of increased SHF+LHF due to sea ice loss on increased surface melt is debated due to the katabatic winds blocking onshore flow (Noël et al. 2014). The results found here indicate the mixing of the katabatic winds with the anomalous warm onshore flow. It is important to acknowledge that the melt response may be highly dependent on the background state of the GrIS. For a warmer GrIS (e.g., due to global warming), the impact of sea ice loss on GrIS melting through SHF+LHF might be higher (Franco et al. 2013; Sellevold and Vizcaino 2020; Muntjewerf et al. 2020b).

Regional enhancement of the Z$_{500}$ over Baffin bay and Greenland occurs in response to the sea ice forcing. A similar but stronger circulation pattern is connected to present-day elevated surface melt of the GrIS (Hanna et al. 2016; Delhasse et al. 2018). We find that this circulation pattern increases onshore advection of heat and moisture in the northwest of GrIS, and reduces it in the southwest. This affects precipitation, with an increase in the northwest and a decrease in the southwest. Further, this increase in Z$_{500}$ is connected with an increase in blocking in northwest Greenland. The increased blocking in this region was also reported by Liu et al. (2016), albeit with a different blocking metric. From our results, we hypothesize that the increase in Z$_{500}$ is triggered by deep warming over the Baffin Bay through high vertical winds (compared to horizontal winds) and high turbulent flow around the GrIS.

Fully coupled CMIP6 simulations (i.e., with an active ocean and sea ice component) show decreases in blocking over Greenland (Delhasse et al. 2021), in opposition to our results presented here. We hypothesize that cooling of sea surface temperatures in the North Atlantic (Sellevold and Vizcaino 2020), due to the projected slowdown of the North Atlantic Meridional Overturning Circulation in the fully coupled models, causes the decrease in Greenland blocking. Our results suggest that future reduced Arctic sea ice would partially counteract the projected reduction in the Greenland blocking index. However, the NAMOC decrease will likely be the dominant influence of change in the Greenland blocking index.

## Conclusions

In our simulations, sea ice loss and increased SSTs warm the Arctic surface and atmosphere in both winter and summer. This Arctic amplification intensifies the hydrological cycle over the GrIS, with 23 ± 33 Gt year$^{-1}$ of increased accumulation in winter and 33 ± 64 Gt year$^{-1}$ of increased ablation in summer, for a sea ice loss corresponding to 2 $^{\circ }$C of global warming as simulated by CMIP5 models.

Such sea ice loss also causes up to 15 m of regional enhancement of the 500 hPa geopotential heights over the GrIS. Recent unprecedented GrIS melt increases have also been partially attributed to regional enhancement of the 500 hPa geopotential heights over the GrIS, with associated atmospheric blocking, anomalous warm wind, and clearer skies (Hofer et al. 2017).

## Supplementary Information

Below is the link to the electronic supplementary material.

Supplementary file1 (PDF 3420 KB)
