# Supplementary material for: Influence of Arctic sea-ice loss on the Greenland ice sheet climate
Source: Clim Dyn. 2021 Jul 29;58(1-2):179–93. doi: 10.1007/s00382-021-05897-4 (PMC8791894; doi:10.1007/s00382-021-05897-4)
Supplement: Supplementary file 1 — Supplementary file1 (PDF 3420 KB) [file 382_2021_5897_MOESM1_ESM.pdf]

6 Supplementary figures

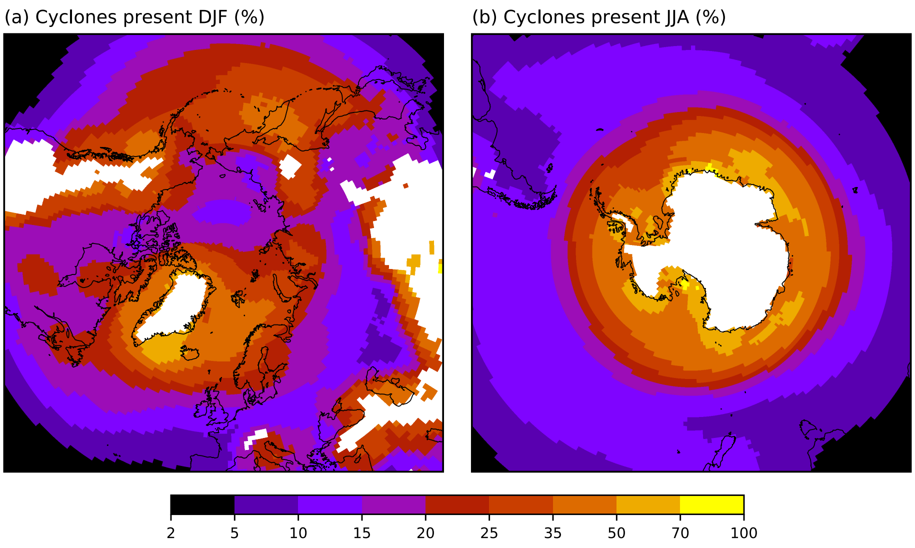

Fig. S1 The amount of time where a cyclone is present in a grid cell (%) in a) DJF and b) JJA.

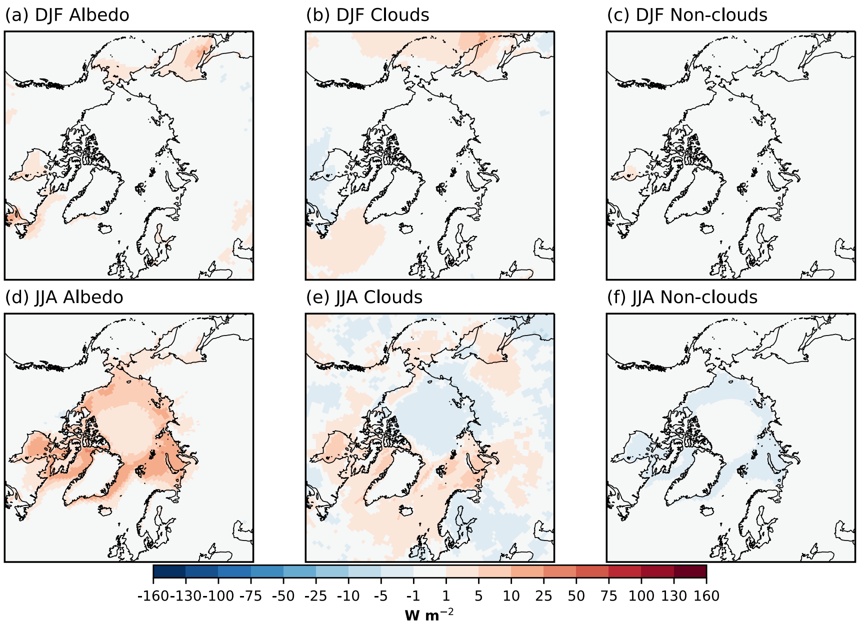

Fig. S2 SW_{net} anomalies explained by changes in (a,d) surface albedo [W m^{-2}], (b,e) clouds [W m^{-2}], and (c,f) non-clouds (e.g., water vapor) [W m^{-2}], using the approximate partial radiative perturbation method (Taylor et al, 2007). Upper and lower rows correspond to winter and summer, respectively.

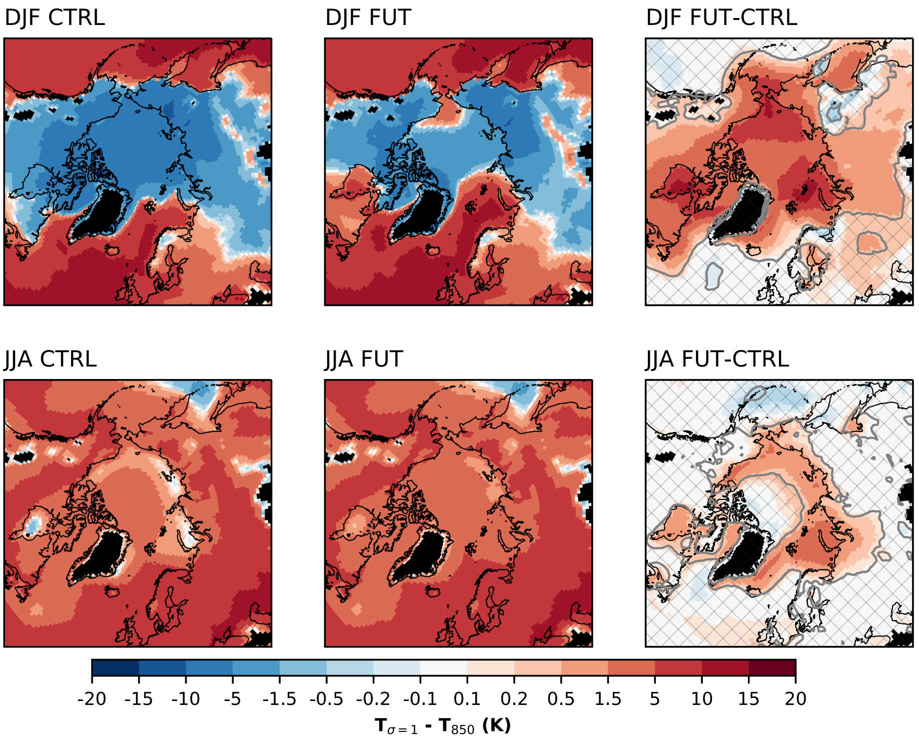

Fig. S3 Temperature difference between the lowest atmospheric model level ($\sigma = 1$) and 850 hPa level (T_{850}) in K for (a,d) CTRL, (b,e) FUT, and (e,f) the difference FUT-CTRL. The upper row shows DJF averages while the lower row shows JJA averages. Patched areas enclosed by the grey contour lines indicate non-significant differences at the 95% level. Black colored areas indicate not-a-number, due to intersection with the 850 hPa surface.
